# Identification of a novel mechanism of action of bovine IgG antibodies specific for *Staphylococcus aureus*

**DOI:** 10.1186/s13567-018-0517-y

**Published:** 2018-02-26

**Authors:** Mutsumi Furukawa, Hiroshi Yoneyama, Eiji Hata, Hidetomo Iwano, Hidetoshi Higuchi, Tasuke Ando, Mika Sato, Tomohito Hayashi, Yoshio Kiku, Yuya Nagasawa, Kanae Niimi, Katsuki Usami, Kumiko Ito, Kouichi Watanabe, Tomonori Nochi, Hisashi Aso

**Affiliations:** 1 International Education and Research Center for Food and Agricultural Immunology, Graduate School of Agricultural Science, Tohoku University, Sendai, Miyagi 980-0845 Japan; 2 National Institute of Animal Health, National Agriculture and Food Research Organization, Sapporo, Hokkaido 062-0045 Japan; 3 School of Veterinary Medicine, Rakuno Gakuen University, Ebetsu, Hokkaido 069-8501 Japan; 4 International Research and Development Center for Mucosal Vaccine, The University of Tokyo, Tokyo, 108-8639 Japan

## Abstract

*Staphylococcus aureus* is a major pathogen that causes subclinical mastitis associated with huge economic losses to the dairy industry. A few vaccines for bovine mastitis are available, and they are expected to induce the production of *S. aureus*-specific antibodies that prevent bacterial adherence to host cells or promote opsonization by phagocytes. However, the efficacy of such vaccines are still under debate; therefore, further research focusing on improving the current vaccines by seeking additional mechanisms of action is required to reduce economic losses due to mastitis in the dairy industry. Here, we generated *S. aureus*-specific bovine IgG antibodies (anti-*S. aureus*) that directly inhibited bacterial growth in vitro. Inhibition depended on specificity for anti-*S. aureus*, not the interaction between Protein A and the fragment crystallizable region of the IgG antibodies or bacterial agglutination. An in vitro culture study using *S. aureus* strain JE2 and its deletion mutant JE2ΔSrtA, which lacks the gene encoding sortase A, revealed that the effect of anti-*S. aureus* was sortase-A-independent. Sortase A is involved in the synthesis of cell-wall-associated proteins. Thus, other surface molecules, such as membrane proteins, cell surface polysaccharides, or both, may trigger the inhibition of bacterial growth by anti-*S. aureus*. Together, our findings contribute insights into developing new strategies to further improve the available mastitis vaccine by designing a novel antigen on the surface of *S. aureus* to induce inhibitory signals that prevent bacterial growth.

**Electronic supplementary material:**

The online version of this article (10.1186/s13567-018-0517-y) contains supplementary material, which is available to authorized users.

## Introduction

Mastitis is an inflammatory disease caused by infection in mammals, such as dairy cattle, by pathogenic microorganisms, such as *Staphylococcus aureus* [1]. Major economic losses incurred by the dairy industry are associated with mastitis [2]. Once *S. aureus* infects the mammary gland of dairy cattle, it can infiltrate deep into the tissue and survive elimination by phagocytic cells, such as macrophages [3]. Therefore, although antibiotics have been used to treat dairy cattle for mastitis caused by *S. aureus*, they do not effectively reach the lesion in most cases, making it difficult to achieve a complete cure [4, 5]. *S. aureus* causes either subclinical or clinical mastitis characterized by abnormal milk containing a large number of somatic cells or by symptoms of per-acute or acute mastitis with pathogen-nonrelated common features, such as swelling, erythema, pain, and perception of heat, respectively [6].

Vaccination is an effective strategy to prevent inflammation, including mastitis. Vaccines, such as STARTVAC^®^ [7–9] and Lysigin^®^ [10], are used globally to protect dairy cattle from mastitis caused by *S. aureus*. These mastitis vaccines comprise killed *S. aureus* or *S. aureus* lysate and act primarily through the induction of antibodies: these may lead to antibody-mediated opsonization and removal of the pathogen through phagocytosis [7–10]. In fact, a recent study addressing the efficacy of STARTVAC^®^ revealed that it evokes the Th2 type of immune response that results in inducing *S. aureus*-specific IgG1 antibody production in not only serum but also milk [9]. Alternatively, it may be possible to inhibit attachment of *S. aureus* to mammary alveoli by binding of the vaccine-induced antibodies to one or more bacterial surface molecules that are involved in adhesion [7–10]. However, the economic loss of the last few decades due to mastitis has remained at approximately $2 billion per year in the US [11, 12]. Therefore, further research is required to enhance the efficiency of mastitis vaccines.

Antibodies possess numerous immunological activities, and their roles are versatile because the animals vaccinated with whole killed bacteria possess polyclonal antibodies with high reactivity for multiple antigens expressed by the immunized bacteria [13]. Further, the feasibility of using antibodies that bind to the bacterial surface to inhibit growth remains to be evaluated. Therefore, inhibiting bacterial growth using antibodies has not been the focus of research compared with that aimed at inhibiting bacterial adhesion to their hosts [14, 15]. However, a complete understanding of the anticipated effects of current vaccines may be important to decrease the morbidity and mortality caused by targeted diseases. Therefore, the purpose of the present study was to examine the role of *S. aureus*-specific antibodies in the inhibition of bacterial growth and define the underlying mechanisms.

Here we generated bovine polyclonal IgG antibodies specific for *S. aureus* (anti-*S. aureus*) by immunizing a Holstein calf with killed *S. aureus*. We demonstrate that the growth of *S. aureus* was inhibited in vitro by anti-*S. aureus*. These results may serve as a foundation for improving current mastitis vaccines to effectively control bacterial growth in vivo.

## Materials and methods

### Bacteria

*S. aureus* (BM1006 [16], SA003 [17] and JE2 [18]), *S. epidermidis* (ATCC14990) [19], *Bacillus atrophaeus* (ATCC9372) [20], and *E. coli* (JM109) [21] were used in the present study. BM1006 was isolated from the bulk milk of dairy cattle, and SA003 originated from the milk of dairy cattle suffering from mastitis. *S. aureus* JE2 is extensively used in the laboratory. A transposon mutant derived from JE2 (NE1787, termed JE2ΔSrtA) [22] was used to investigate sortase-A-dependent cell-wall-associated proteins. The evolutionary relationships among the 16S ribosomal RNA genes of 21 bacteria and the Archaea *Pyrococcus horikoshii* (listed in Additional file 1) were inferred using the UPGMA method, and evolutionary distances were calculated using the Maximum Composite Likelihood method of MEGA7 as previously described [23, 24].

### Antibody production

A Holstein calf (5 months old, male) was subcutaneously immunized with formalin-killed *S. aureus* [BM1006, 1.5 × 10^10^ colony-forming units (CFU)] together with TiterMax^®^ Gold (TiterMax) on three occasions at 2-week intervals. One week after the final immunization, serum was collected, and polyclonal IgG antibodies were purified using Protein G Sepharose 4 Fast Flow (GE healthcare). The concentration of IgG antibodies after purification was measured by BCA Protein Assay Kit (Thermo Fisher). The use of the Holstein calf to produce antibodies was performed in accordance with protocols approved by the Institutional Animal Care and Use Committee of Tohoku University.

### Enzyme-linked immunosorbent assay (ELISA)

ELISA were performed to determine the amount and specificity of purified polyclonal bovine IgG antibodies. Briefly, 96-well plates (Nunc) were coated with 2 μg/mL of purified sheep anti-bovine IgG-heavy chain antibodies (Bethyl) overnight at 4 °C. Alternatively, the plates were coated with 5 μg protein/mL of either *S. aureus*, *S. epidermidis*, *B. atrophaeus*, or *E. coli*, each killed with 0.5% (w/v) formalin (Wako). After blocking with 0.05% (v/v) Tween-20 in Tris-buffered saline for 1 h at room temperature (RT), twofold serial dilutions of polyclonal IgG antibodies obtained from the immunized Holstein calf or control bovine IgG antibodies (Sigma-Aldrich) were incubated for 2 h at RT. After washing, HRP-conjugated sheep anti-bovine IgG-heavy chain antibodies diluted 1:10 000 (Bethyl) were treated for 1 h at RT, and the reactions were developed with a TMB microwell peroxidase substrate system (KPL).

### Flow cytometry

Bacteria killed with 0.5% (w/v) of formalin were incubated for 30 min at 4 °C with 1000 μg/mL of either anti-*S. aureus* or control IgG, both of which were conjugated with FITC (Sigma-Aldrich), as described previously [25]. Untreated bacteria were prepared as a control. After washing, the bacteria were analyzed using a BD Accuri C6 Flow Cytometer (BD Bioscience), and the data were analyzed using FlowJo (Digital Biology).

### SDS-PAGE and western blotting

Bacteria were lysed with SDS sample buffer containing 62.5 μM of Tris–HCl (pH 6.8), 2% (w/v) SDS, 10% (v/v) glycerol, 5% (v/v) 2-mercaptoethanol, and 0.02% (w/v) bromophenol blue; the extracts were subjected to SDS-PAGE using a 5–20% e-PAGEL polyacrylamide gel (ATTO). After electrophoresis, the gel was stained with SimplyBlue SafeStain (Invitrogen), or the proteins were transferred to an Immobilon-P membrane (Millipore). The membrane was treated with 10 μg/mL of either the anti-*S. aureus* or control IgG antibody for 1 h at RT, after blocking the membrane with 0.05% (v/v) of Tween-20 in Tris-buffered saline overnight at 4 °C. After washing, the membrane was treated with HRP-conjugated sheep anti-bovine IgG-heavy chain antibodies diluted 1:10 000 (Bethyl) for 1 h at RT, and the reaction was developed using an EzWestLumi plus (ATTO).

### Bacterial culture in vitro

*Staphylococcus aureus* (BM1006, SA003, JE2, and JE2ΔsrtA) and *E. coli* (JM109) were cultured overnight at 37 °C in Trypto-Soya (TS) broth (Nissui), and 40 μL was added to 4 mL of fresh TS broth containing anti-*S. aureus* (10, 100, and 1000 μg/mL) or control IgG (1000 μg/mL) to address the effect of anti-*S. aureus* on the bacterial growth. In some experiments, IgG antibodies in the used medium that originally included anti-*S. aureus* were re-used for the additional in vitro study to test the effect. Size and autofluorescence intensity of *S. aureus* cultured for 0.5, 2, 5, and 24 h in the presence of either anti-*S. aureus* or control IgG were analyzed using a microbial particle counter (Rion). In brief, the culture broth of *S. aureus* was diluted 1:100 000 with distilled water and subjected directly to microbial particle count analysis in which the scattered light and autofluorescence intensity mediated by Riboflavin derived from violet laser-exposed bacteria were measured. The morphology of *S. aureus* cultured for 0.5, 2, 5, and 24 h was analyzed using a scanning electron microscope (SEM). Specifically, the culture medium of *S. aureus* (BM1006) was removed after quick centrifugation for 1 min, and the remaining bacterial pellet was fixed with 2.5% (v/v) glutaraldehyde (Nacalai tesque) in 0.1 M phosphate buffer for 1 h at 4 °C. After washing the bacterial pellet to remove excess fixative, bacteria were suspended using distilled water and affixed to glass slides (Matsunami). The bacteria were then coated with platinum and palladium. Images of *S. aureus* were obtained using an SEM (SU8000, Hitachi) operated at 3.0 kV. Survival of bacterial cells at 0.5, 2, 5, and 24 h after the culture was determined by obtaining CFU. Specifically, aliquots of bacterial broth were collected and seeded on TS agar plates (three replicates) after dilution with saline. To determine the quantitative effect of anti-*S. aureus* in TS broth during bacterial culture, samples were collected at 0.5, 2, 5, and 24 h after the start of culture and filtered using 0.45-μm syringe filters (Advantec) to remove antibodies bound to bacteria. The pass-through solutions were subjected to ELISA analyses to determine both the total amount of IgG antibodies and the titer of *S. aureus*-specific antibodies after the use of antibodies as additive in the *S. aureus* culture. To address those, ELISA analyses were performed using 96-well plates coated with either purified sheep anti-bovine IgG-heavy chain antibodies (Bethyl) or killed *S. aureus* as described above.

### Statistics

Statistical analyses were performed using one-way ANOVA with the Kruskal–Wallis test and two-way ANOVA with Tukey’s multiple comparisons test using Prism 7 software (GraphPad).

## Results

### Generation of bovine IgG antibodies against *S. aureus*

**Figure 1:**
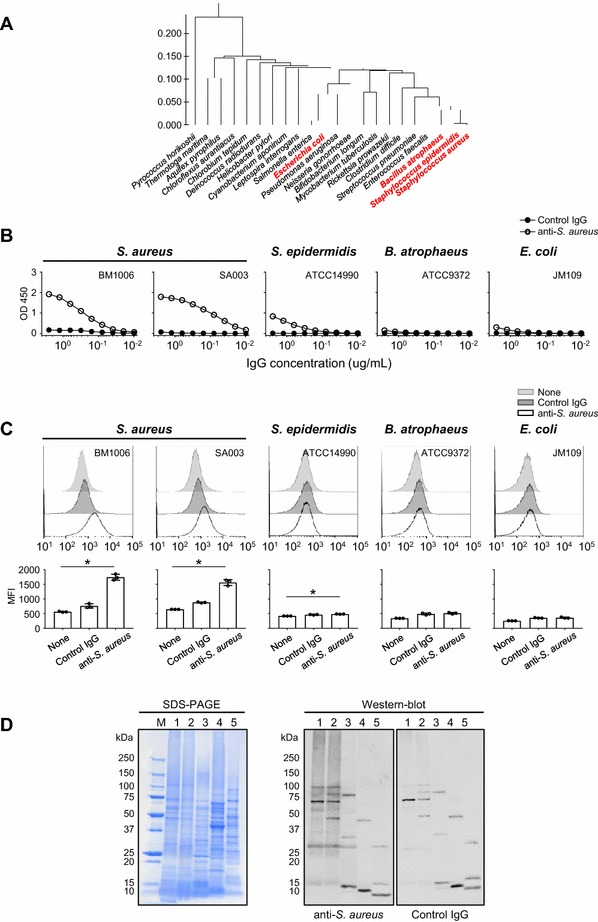
**Generation of anti-*S. aureus*-specific bovine IgG antibodies.** *S. aureus*, *S. epidermidis*, *B. atrophaeus,* and *E. coli* were used to confirm the specificity of antibodies against *S. aureus* (anti-*S. aureus*) generated by immunization of a Holstein dairy cow with killed *S. aureus*. **A** Genetic similarity of the four species of bacteria used in this study and other well-characterized bacteria is shown as a phylogenetic tree. **B** ELISA analyses show that anti-*S. aureus* was highly reactive with two strains of *S. aureus* (BM1006 and SA003) and less reactive *S. epidermidis*. In contrast, little reactivity was detected when anti-*S. aureus* was reacted with *B. atrophaeus* and *E. coli*. **C** Flow cytometric analyses detected similar reactivities, respectively. **D** SDS-PAGE and western blot analyses revealed that anti-*S. aureus* reacted with *S. aureus*-specific molecules with a broad range of molecular masses. M: molecular marker, 1: *S. aureus* (BM1006), 2: *S. aureus* (SA003), 3: *S. epidermidis*, 4: *B. atrophaeus*, 5: *E. coli*. Three separate experiments were performed, and the data represent the mean ± standard error of the mean. *p < 0.05.

Since mastitis vaccines were not commercialized in Japan when we started this study, we first performed an immunization study with killed *S. aureus* prepared in our laboratory to reproduce the current mastitis vaccine trials that have been tested outside Japan. Specifically, a Holstein calf was subcutaneously immunized with killed *S. aureus* BM1006 that was isolated from bulk milk of dairy cattle and IgG antibodies were purified from the serum collected from the immunized calf. Antibody specificity was confirmed using an ELISA and the test antigens as follows: *S. aureus*, *S. epidermidis*, *B. atrophaeus*, and *E. coli*. Figure 1A shows a phylogenetic tree displaying the genetic similarity among bacteria used in this study and other well-characterized bacteria. The IgG antibodies reacted with BM1006 in a concentration-dependent manner; however, the control bovine IgG antibodies were not detectably reactive (Figure 1B). We therefore defined the immunized and control IgG antibodies as anti-*S. aureus* and control IgG, respectively. Anti-*S. aureus* strongly reacted with *S. aureus* SA003 (Figure 1B). *S. epidermidis*, which is phylogenetically closely related to *S. aureus*, exhibited little reactivity with anti-*S. aureus.* Further, *B. atrophaeus* and *E. coli* (members of Firmicutes and Proteobacteria, respectively) showed little or no reactivity (Figure 1B). To determine the specificity of anti-*S. aureus*, we performed flow cytometric analyses. Consistent with the ELISA results, anti-*S. aureus*, reacted with BM1006 and SA003 but not with the control IgG (Figure 1C). Weak reactivity was detected when *S. epidermidis* was incubated with anti-*S. aureus*, but reactivity with *B. atrophaeus* or *E. coli* was undetectable (Figure 1C). SDS-PAGE and western blot analyses revealed that molecules with a broad range of molecular masses that are specifically expressed by *S. aureus* but not by *S. epidermidis*, *B. atrophaeus*, or *E. coli* were recognized by anti-*S. aureus* (Figure 1D). Some bands detected by both anti-*S. aureus* and control IgG may be due to the reactivity of natural IgG antibodies or non-specificity (Figure 1D).

### Growth in vitro of *S. aureus* is inhibited by anti-*S. aureus*

**Figure 2:**
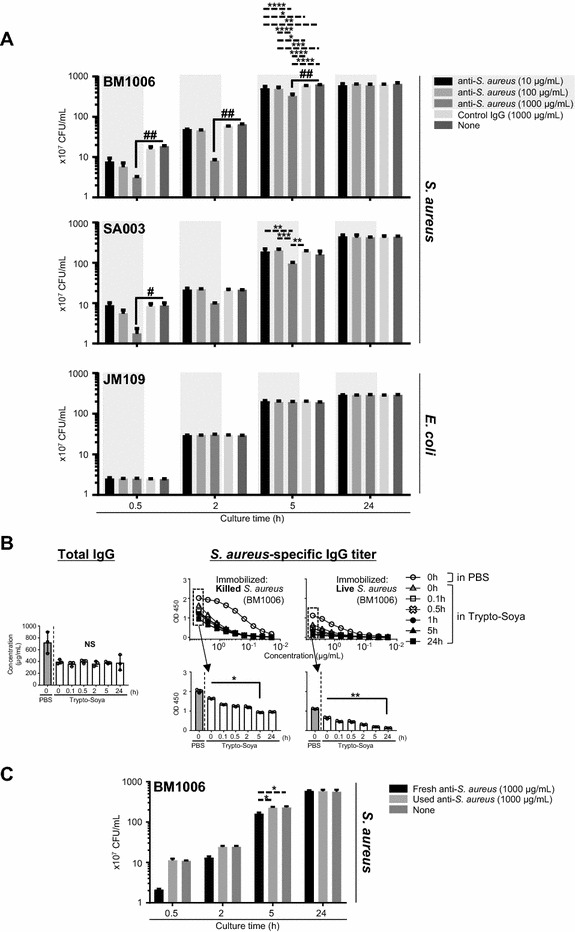
**Inhibition of the growth in culture of** ***S. aureus*** **in the presence of anti-*S. aureus.*** The neutralizing effects in vitro of anti-*S. aureus* on the growth of cultures of *S. aureus* and *E. coli*. **A** Growth of *S. aureus* (BM1006 and SA003 was inhibited in the presence of anti-*S. aureus*, whereas the growth of *E. coli* (JM109) was not detectably inhibited. **B** The amount of uncoupled total IgG remained constant in the culture broth during the overnight culture of *S. aureus* (BM1006). In contrast, residual killed and live *S. aureus*-specific IgG titers gradually decreased during the overnight culture. **C** The anti-*S. aureus* that was used in the overnight culture of *S. aureus* did not inhibit bacterial growth when reused. Three separate experiments were performed, and the data represent mean ± standard error of the mean. The *p* values calculated using one-way or two-way factorial analyses of variance are ^#^*p* < 0.05 and ^##^*p* < 0.01 or *p < 0.05; **p < 0.01; ***p < 0.001; and ****p < 0.0001, respectively.

We found that the growth of *S. aureus* BM1006 and SA003 was delayed in the presence of anti-*S. aureus* but was undetectable when control IgG was added to the cultures (Figure 2A). Bacterial growth in vitro was inhibited by a relatively high concentration of anti-*S. aureus* (1000 µg/mL), although the overall effect was concentration-dependent (Figure 2A). Moreover, anti-*S. aureus* did not detectably inhibit the growth of *E. coli* (Figure 2A). These results indicate that the antigen-specific binding of anti-*S. aureus* (not nonspecific binding, such as the interaction between Protein A on the surface of *S. aureus* and the Fc region of IgG antibodies) may be required for inhibiting bacterial growth in vitro. However, the growth of *S. aureus* plateaued when cultured overnight with or without the anti-*S. aureus* (Figure 2A), indicating that the inhibitory effect of anti-*S. aureus* was not sustained during overnight culture. Therefore, we performed a study using an ELISA to determine the change of anti-*S. aureus* concentrations during an overnight incubation of the culture with 1000 μg/mL anti-*S. aureus*. The concentration of total IgG antibodies remained constant, although the concentration of total IgG in PBS (Figure 2B) was 721.5 ± 181.2 μg/mL (not 1000 μg/mL). This may be explained by the use of different assays. For example, the nonspecific BCA protein assay was used basically to measure the purified IgG. Further, a lower concentration was calculated when anti-*S. aureus* was suspended in TS medium (395.1 ± 37.5 μg/mL 0 h after culture), suggesting that the bovine IgG ELISA system was affected by the solvent. Nevertheless, there were no significant differences in the concentrations of total IgG antibodies in the culture broth during the overnight culture. In contrast, the *S. aureus*-specific IgG titer of the culture broth gradually decreased during the 24 h culture when the assay used ELISA plates immobilized with killed or live *S. aureus* (Figure 2B). Inhibition was not detected when the anti-*S. aureus* used for overnight culture was reused for the in vitro bacterial culture study, consistent with the undetectable antibody titer specific for live *S. aureus* 24 h after the culture (Figure 2C). The anti-*S. aureus* is a polyclonal antibody that likely recognized different epitopes. Therefore, these results indicate that a small amount of antibodies in the anti-*S. aureus* preparation reacted with *S. aureus* and inhibited bacterial growth in vitro.

### Antibody-mediated growth inhibition of *S. aureus* is not due to bacterial agglutination

**Figure 3:**
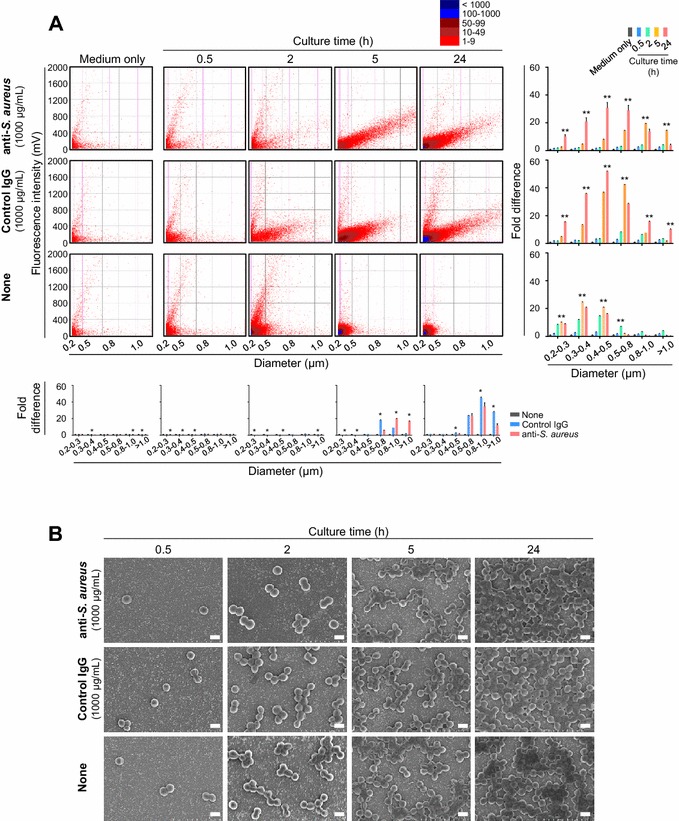
**Alteration of bacterial features other than growth or agglutination in** ***S. aureus*** **is caused by bovine IgG antibodies regardless of antigen–antibody reaction.** Bacterial features of *S. aureus* in culture with anti-*S. aureus* were determined using a microbial particle counter and a scanning electron microscopy. **A** Size and autofluorescence intensity that can be observed by the microbial particle counter were increased gradually when *S. aureus* was cultured with either anti-*S. aureus* or control IgG whereas those bacterial features were not altered when *S. aureus* was cultured without any antibody treatment. The analyzer show dots that indicate the detected particles, including mostly bacteria and some debris in the medium. The difference in the color of the dot indicates the difference in the number of particles detected. Bar graphs indicate the fold difference in the number of particles observed when the number detected in medium alone is indicated as 1. Right three panels present comparisons at different culture time under same culture medium condition. Bottom five panels present comparisons at different culture medium condition at same culture time. **B** Scanning electron microscopy of *S. aureus* showed that there is no difference in morphological characteristics (not number) of *S. aureus* regardless of culture conditions. Three separate experiments were performed, and representative data are shown. The *p* values calculated using one-way factorial analyses of variance in each diameter in comparison with different culture time in the same culture medium (shown on the right three panels) or different culture medium in the same culture time (shown on the bottom five panes) are ^#^*p* < 0.05 and **p < 0.01 (vs medium only or None).

Considering that CFU do not indicate the bacterial number, the actual number of *S. aureus* may be different from the CFU number in cases where one colony does not necessarily arise from one bacterium upon bacterial agglutination. To dispel this concern, we directly measured the diameter of *S. aureus* during the in vitro culture using a microbial particle counter, which allows us to investigate the size and autofluorescence intensity of particles in the sample. We found that the number of *S. aureus* whose size and autofluorescence intensity increased was gradually elevated when cultured in the presence of both anti-*S. aureus* and control IgG (Figure 3A and Additional file 2). In contrast, such altered *S. aureus* was rarely observed when cultured without any supplementation (Figure 3A and Additional file 2). We next performed an SEM analysis to directly observe the morphological characteristics of *S. aureus* in the in vitro culture. Consistent with the well-known feature of *S. aureus*, we observed the typical structure of *S. aureus* in the form of a grape-like cluster when cultured with neither anti-*S. aureus* nor control IgG (Figure 3B). Importantly, the morphological characteristics (not the bacterial number) seemed to remain unchanged, although *S. aureus* was cultured in the presence of anti-*S. aureus* or control IgG (Figure 3B). Because the fragment crystallizable (Fc) region of IgG antibodies binds to protein A on the surface of *S. aureus* [26], these results suggest that, regardless of antibody specificities, the IgG antibodies that associate with Protein A may alter bacterial features (other than growth or agglutination) that can be detected as increased size and autofluorescence intensity, even though the reactivity between Fc region of IgG antibodies and Protein A was almost undetectable in our previous analyses (Figures 1B–D). Nevertheless, it should be emphasized that these phenomena observed were not due to bacterial agglutination; thus, the inhibition of bacterial growth caused by anti-*S. aureus* could be mediated in a specific manner of antigen–antibody reaction.

### Reactivity of anti-*S. aureus* to molecules expressed specifically by *S. aureus* (not commonly expressed by closely related bacteria) are mainly involved in the inhibition of bacterial growth in vitro

**Figure 4:**
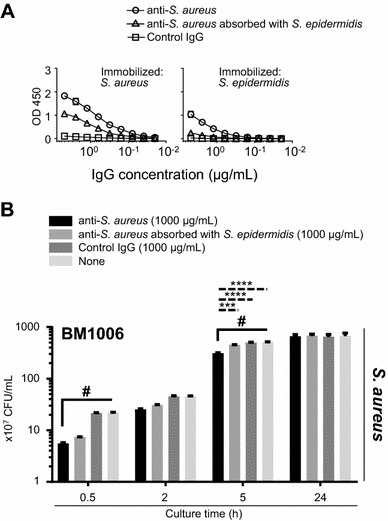
**Involvement of** ***S. aureus*-specific molecules in anti-*S. aureus*-induced growth inhibition.** ELISA analyses and in vitro bacterial culture studies confirmed the bacterial specificity of growth inhibition induced by anti-*S. aureus*. **A** After absorption of anti-*S. aureus* with *S. epidermidis*, the *S. aureus*-specific antibody titer was slightly decreased compared with that of the original anti-*S. aureus*. In contrast, little reactivity of anti-*S. aureus* with *S. epidermidis* was detected after absorption. **B** Anti-*S. aureus* absorbed with *S. epidermidis* did not inhibit the growth of *S. aureus*. Three separate experiments were performed, and the data represent the mean ± standard error of the mean. The *p* values calculated using one-way or two-way factorial analyses of variance are ^#^*p* < 0.05 or ***p < 0.001 and ****p < 0.0001, respectively.

Anti-*S. aureus* slightly reacted with *S. epidermidis* (Figures 1B and C). Therefore, we hypothesized that antibody recognition of common molecules expressed by *Staphylococcus* species (e.g., *S. aureus* and *S. epidermidis*) was involved in the inhibition of bacterial growth in vitro. We thus incubated anti-*S. aureus* with *S. epidermidis* and collected the unbound antibodies by centrifugation to remove reactivity to molecules that are expressed by *S. epidermidis*. ELISA analysis revealed that anti-*S. aureus* absorbed with *S. epidermidis* lost most of its reactivity with *S. epidermidis* (Figure 4A). In contrast, although the reactivity to *S. aureus* was slightly lower when compared with the original anti-*S. aureus*, it was detected after absorption with *S. epidermidis* (Figure 4A). Moreover, when anti-*S. aureus* was absorbed with *S. epidermidis*, little or no effect was observed on the ability of anti-*S. aureus* to inhibit bacterial growth (Figure 4B). These results suggest that among the numerous epitopes recognized by anti-*S. aureus*, its reactivity with specific molecules on *S. aureus* (not common molecules on both *S. aureus* and *S. epidermidis*) are largely responsible for triggering the inhibition of bacterial growth.

### Antibody recognition of sortase-A-dependent cell-wall-associated proteins is not associated with the capacity of anti-*S. aureus* to inhibit bacterial growth in vitro

**Figure 5:**
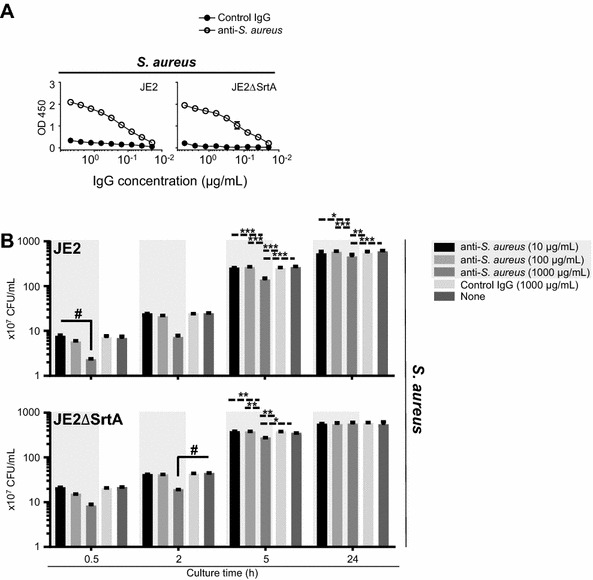
**Dispensable role of sortase-A-dependent cell-wall-associated proteins associated with anti-*S. aureus*-induced growth inhibition.** The involvement of antibody recognition of sortase-A-dependent cell-wall-associated proteins in the growth inhibition of *S. aureus* was addressed using *S. aureus* strains JE2 and JE2ΔSrtA. **A** ELISA analyses revealed that anti-*S. aureus* reacted equally with JE2 and JE2ΔSrtA. **B** The growth of JE2 and JE2ΔSrtA was delayed when they were cultured with anti-*S. aureus*. Three separate experiments were performed, and the data represent the mean ± standard error of the mean. The *p* values calculated using one-way or two-way factorial analyses of variance are ^#^*p* < 0.05 or *p < 0.05; **p < 0.01 and ***p < 0.001, respectively.

Cell-wall-associated proteins that bind peptidoglycans produced by *S. aureus* are involved in pathogenicity and are therefore considered targets of an *S. aureus* vaccine [27]. Hence, we asked whether the recognition of anti-*S. aureus* to cell-wall-associated proteins inhibited bacterial growth in vitro. We used JE2, which is a plasmid-cured derivative of strain LAC, originally isolated from a human, and its deletion mutant that lacks the gene (JE2ΔSrtA) encoding sortase A because sortase A acts on many cell-wall-associated proteins to link them to peptidoglycans [28]. We found that the reactivities of anti-*S. aureus* with JE2 and JE2ΔSrtA were comparable (Figure 5A). Further, there was no significant difference between the reactivity with BM1006 or SA003 (Figure 1B). However, we did not exclude the possibility that certain sortase-A-dependent cell-wall-associated proteins were recognized by anti-*S. aureus*. Moreover, the SDS-PAGE and western blot data suggest that anti-*S. aureus* reacted with multiple surface molecules, such as membrane proteins that are not substrates of sortase, cell wall polysaccharides, or both (Figure 1D). Therefore, we addressed the possibility of sortase-A-independent growth inhibition induced by anti-*S. aureus* as indicated by the results of the in vitro bacterial culture study using JE2 and JE2ΔSrtA. We found that the growth of JE2 and JE2ΔSrtA was inhibited when they were cultured in the presence of anti-*S. aureus* (Figure 5B), indicating that the inhibition of bacterial growth by anti-*S. aureus* was sortase-A-independent. Together, our results provide new insights into devising strategies for developing an *S. aureus* vaccine that induces signals that inhibit bacterial growth or blocks absorption of nutrients that are essential for bacterial growth by vaccine-induced antibodies specific for surface molecules that have not been previously identified.

## Discussion

Increasing the efficacy of mastitis vaccines is a global challenge in the field of dairy science [29]. We focused on investigating the inhibitory effect on bacterial growth by specific bovine IgG antibodies produced by vaccination of a Holstein calf with *S. aureus* killed with formalin. *S. aureus* is frequently found in the nose, respiratory tract, and skin of animals, including humans and livestock [30–32]. *S. aureus* is not always pathogenic, but occasionally causes infectious diseases, such as pneumonia, infective endocarditis, sepsis, osteomyelitis, and mastitis [33]. The molecular mechanisms of *S. aureus* infection is under investigation; however, several molecules expressed by *S. aureus* are involved in pathogenesis [34]. For example, exotoxin (staphylococcal enterotoxins and toxic shock syndrome toxin-1) [35, 36], binding molecules (clumping factors and fibronectin binding proteins) [37], and receptors (iron acquisition factors and manganese uptake receptors) [38, 39] are virulence factors involved in the pathogenesis. Therefore, numerous studies of virulence molecules were performed to develop an effective vaccine to protect humans as well as dairy cattle from infectious diseases caused by *S. aureus*. However, despite numerous human clinical trials, most studies have failed to demonstrate the efficacy of *S. aureus* vaccines [33]. Moreover, efforts to develop *S. aureus* vaccines for dairy cattle lag behind those of humans [33]. Antibiotics have been widely used in dairy cattle to treat subclinical mastitis caused by *S. aureus*; however, this may increase the risk of selecting for antibiotic-resistant bacteria, including MRSA [40]. Therefore, a vaccine strategy, which is independent of antibiotic resistance, to control the growth of *S. aureus* is important to prevent the distribution of antibiotic-resistant *S. aureus*. Nevertheless, it is noteworthy that the bovine mastitis vaccine STARTVAC^®^ elicits the production of *S. aureus*-specific bovine IgG antibodies that inhibit the formation of biofilms and facilitates opsonization [7–9].

We show here, for the first time to our knowledge, that *S. aureus*-specific bovine IgG antibodies directly inhibited the growth of *S. aureus* without supplementation (e.g., complement) in an in vitro culture system. The effects of anti-*S. aureus* were similar among strains, because the growth of JE2, which is a methicillin‐resistant *S. aureus* (MRSA) [41], was equivalently inhibited by treatment with anti-*S. aureus* compared with BM1006 and SA003, which are non-MRSA strains isolated from the milk of dairy cattle (Figures 2–4).

Gram-positive bacteria like *S. aureus* develop a thick peptidoglycan layer with cell-wall-associated proteins that are linked to peptidoglycans [42]. The molecular mechanism of the synthesis of a dozen cell-wall-associated proteins in the peptidoglycan layer requires cleavage by sortase A of the peptide bond between Thr and Gly residues in the LPXTG motif, which is a consensus motif present within the C-terminus of sortase substrate proteins [27]. Sortase A produces alternative bonds between the carboxyl group of Thr and the amino groups of peptidoglycans [28]. Sortase-A-dependent cell-wall-associated proteins play important roles in infection by *S. aureus* and mediate adhesion to host cells and evasion of the pathogen of the host’s immune system [37]. In addition, such proteins are also involved in absorbing nutrients, such as iron [38]. However, our results acquired using *S. aureus* JE2ΔSrtA show that the inhibition of bacterial growth by anti-*S. aureus* was sortase-A-independent (Figure 5). These findings suggest that potential target molecules, which may be involved in inhibiting bacterial growth through recognition of anti-*S. aureus*, may not include cell-wall-associated proteins that are substrates of sortase A. The surfaces of gram-positive bacteria such as *S. aureus* include four major proteins (e.g., membrane proteins, lipoproteins, LPXTG-like proteins, and cell-wall-associated proteins) [42–44]. Further, cell-wall-associated proteins and extracellular polysaccharides play an important role in host-parasite interactions. Therefore, this information, coupled with our present results, led us to hypothesize that membrane proteins, which are not sortase substrates, polysaccharides, or both, may stimulate a signaling cascade that inhibits bacterial growth through the recognition of anti-*S. aureus*.

Using the microbial particle counter, we detected that the size and autofluorescence intensity of *S. aureus* increased gradually when the bacteria was cultured with IgG antibodies regardless of the specificity. Nevertheless, the morphological and microbiological characteristics of *S. aureus* cultured with either anti-*S. aureus* or control IgG were identical to those of bacteria cultured without any supplementation. Thus far, three possibilities (i.e., increase in bacterial size and alteration of bacterial morphology or refractive index) have been considered as factors that increase scattered light emitted by bacteria [45]. However, in SEM analysis no changes (except the reduction of bacterial number) were observed despite the addition of anti-*S. aureus* to the *S. aureus* culture. These results suggest that the refractive index of scattered light from *S. aureus* may be altered when the Fc region of IgG antibodies bind to Protein A on the surface of *S. aureus*. Another possible hypothesis that causes the increase of autofluorescence intensity may be because of the increase in the amount or quantum yield of Riboflavin, which is a bacteria-derived fluorescence molecule detected by the microbial particle counter used, by the binding of the Fc region of IgG antibodies to Protein A.

Determining the optimal concentration of antibodies required for inhibition of bacterial growth is difficult. For example, we used a concentration of anti-*S. aureus* that was extremely high (1000 μg/mL), although its effects were concentration-dependent (Figures 2–5). Our results were consistent with the past studies showing the efficacy of egg yolk IgY antibodies specific for *S. aureus* to inhibit bacterial growth [46, 47]. Also, others have shown that monoclonal antibody specific to glucosaminidase, which is an enzyme involved in cell wall digestion during binary fission, can induce the abnormality of bacterial survival [48, 49]. Although the optimal concentration of anti-glucosaminidase monoclonal antibody that is sufficient for inhibiting bacterial growth has not yet been addressed, we assumed that it was not high because of high antigenic specificity of monoclonal antibody to single antigen compared with that of polyclonal antibodies to multiple antigens. This may be explained by the use of polyclonal antibodies that include nonspecific antibodies. However, our goal is to identify molecules that can be used as novel antigens to develop a mastitis vaccine and not to utilize the anti-*S. aureus* as passive prophylaxis. Therefore, our current efforts focus on identifying target molecules that stimulate inhibitory signals to suppress bacterial growth by binding vaccine-induced antibodies. Western blot analysis detected several candidate molecules recognized by anti-*S. aureus* (Figure 1D), which must be identified. Further, advances in microbiological research led to the creation of a transposon mutant library of *S. aureus*, which may facilitate efforts to identify target molecules, such as those not related to sortase substrates, cell wall polysaccharides, or both, in our anti-*S. aureus* in vitro culture system. Taken together, our findings have significant potential to further improve mastitis vaccines through antibody-mediated growth control of target bacteria.

## Additional files

**Additional file 1.** **Characteristics of 21 bacteria and Archaea** ***Pyrococcus horikoshii*** **selected for studying the evolutionary relationships based on the sequence of 16s rRNA.**

**Additional file 2.** **The numbers of bacterial particles detected using a microbial particle counter.**

## Abbreviations

ANOVA: analysis of variance
ELISA: enzyme-linked immunosorbent assay
Fc: fragment crystallizable
FITC: fluorescein isothiocyanate
IgG: immunoglobulin G
MRSA: methicillin‐resistant *S. aureus*
PBS: phosphate buffered saline
RT: room temperature
SDS: sodium lauryl sulfate
SEM: standard error of the mean
TS: Trypto-Soya
